# Improving the Quality of Brain PET Images Using MR‐Guided PET Reconstruction Technique With Integrated PET/MR

**DOI:** 10.1002/hbm.70292

**Published:** 2025-08-04

**Authors:** Hongda Shao, Gan Huang, Yue Wang, Yan Zhang, Qiaoyi Xue, Jiaxin Hao, Jianjun Liu

**Affiliations:** ^1^ Department of Nuclear Medicine Ren Ji Hospital, Shanghai Jiao Tong University School of Medicine Shanghai China; ^2^ Central Research Institute UIH Group Shanghai China

**Keywords:** brain PET/MR, image quality, MR‐guided PET reconstruction, PET/MR

## Abstract

To evaluate the performance of an MR‐guided PET reconstruction method in enhancing the image quality of brain PET images acquired with an integrated PET/MR system. We implemented an MR‐guided PET reconstruction (MRg) algorithm in an integrated PET/MR system and conducted both phantom and clinical studies. The PET images of a Hoffman phantom were reconstructed using the MRg algorithm and routine Ordered Subsets Expectation Maximization (OSEM) algorithm. Image quality metrics were calculated to investigate the dose effects, number of iterations, and penalization factor (*β*). In the clinical study, the PET images of 30 patients without brain lesions, two patients with diffuse large B cell lymphoma (DLBCL), 18 patients with Parkinson's disease (PD) and one patient with cavernous angioma were reconstructed using MRg and OSEM, respectively. Qualitative and quantitative assessments were performed. In the phantom study, MRg images demonstrated improved image quality metrics (higher contrast and lower variability) compared to conventional OSEM, particularly at lower doses. The penalization factor β=2 maximized the advantages of MRg. In the clinical study of patients without brain lesions, MRg significantly outperformed OSEM in image quality evaluation. For patients with DLBCL, the lesions' signal/contrast‐to‐background/noise ratios exhibited significant improvements using the MRg. For patients with PD, the standardized uptake value ratio and contrast‐to‐noise ratio of bilateral putamen were significantly improved by MRg. The MRg has convincingly demonstrated significant advantages over the conventional OSEM in brain PET images, indicating great potential in clinical routines for brain PET/MR examinations.

## Graphical abstract

The PET list‐mode data was processed with OSEM algorithm and MR‐guided reconstruction (MRg) based on T1‐weighted MR images (TIWI). Several quantitative parameters were calculated to compare the image quality for conventional OSEM, OSEM_GF, and MRg reconstructions with varying parameters (θ = 0.5, 1, 2). The MRg PET reconstruction demonstrates significant advantages over conventional OSEM in brain PET/MR imaging, highlighting its clinical potential.

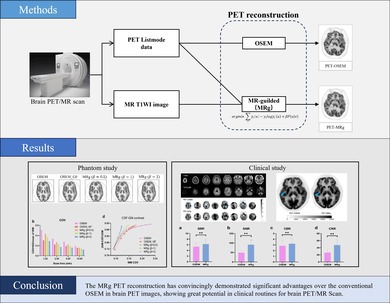

**Summary**

- MRg images demonstrated superior image quality metrics compared to OSEM in the phantom study, particularly at lower doses. The penalization factor 𝛽 = 2 was found to maximize the benefits of MRg.
- For brain PET images of patients with or without brain lesions, MRg outperformed OSEM in image quality evaluation. The improved image quality provided by MRg has the potential to enhance the diagnostic accuracy for various neurological diseases.

## 1 Introduction

With the continuous progress of medical technology, the integration of positron emission tomography (PET) and magnetic resonance imaging (MR) has emerged as an essential tool in modern medical imaging diagnosis (Seifert et al. 2022). The integrated PET/MR system combines the advantages of PET in metabolic imaging with the strengths of MR in anatomical structural and functional imaging. This combination provides clinicians with more comprehensive and accurate diagnostic information, leading to its widespread application in various neurological diseases (Mirshahvalad et al. 2022, 2024). In studying brain tumors, fluorodeoxyglucose PET (FDG‐PET) quantification provides information concerning the degree of malignancy and patient prognosis (Hirata et al. 2012; Coleman et al. 1991). For neurodegenerative diseases, FDG‐PET images play a crucial role in facilitating the differential diagnosis of various subtypes within the Parkinsonian syndromes and assessing the clinical manifestations of cognitive decline (Meyer et al. 2017; Matthews et al. 2018). However, the quality of PET images from the integrated PET/MR scanner is often affected by various factors such as noise, artefacts, and inappropriate attenuation correction (Lindemann et al. 2024; Han et al. 2020). In addition, the relatively poor spatial resolution of PET images can lead to partial volume effects (PVEs), such as the spillover of small high‐intensity regions to neighboring voxels, introducing bias in regional quantification (Thomas et al. 2011; Guedj et al. 2022; Baete et al. 2004; Goffin et al. 2010).

In recent years, MR‐guided PET reconstruction (MRg) has attracted widespread academic attention (Corda‐D'Incan et al. 2020; Mehranian et al. 2019; Deidda et al. 2017; Belzunce et al. 2019; Knoll et al. 2017; Bowsher et al. 2004; Bai et al. 2013). Magnetic resonance imaging (MRI) provides excellent soft tissue contrast, and MR information can be incorporated into the PET reconstruction process through the addition of a regularizing term in either a Bayesian maximum a posteriori (MAPEM) or kernel method (KEM) framework (Bai et al. 2013; Wang and Qi 2015). Various penalization methods have been studied to reduce smoothing across genuine boundaries. These methods can effectively reduce image noise and artifacts enhance image contrast and clarity, and maintain image stability under low‐dose conditions (Bland et al. 2019).

Despite the potential benefits of anatomically‐guided PET reconstruction, its clinical experience remains limited (Schramm et al. 2021). Goffin et al. reported favorable outcomes of anatomically‐guided PET reconstruction in epilepsy (Goffin et al. 2010). However, in that study, the PET and the MR images were acquired separately, requiring registration of PET and MR images from two different scanners, which greatly limited its clinical application. The advent of integrated PET/MR has enabled simultaneous acquisition of PET and MR images, holding promise for the broader clinical application of the MRg algorithms.

This study's purpose was to implement an MR‐guided PET reconstruction algorithm in an integrated PET/MR system and to evaluate its effectiveness in improving the image quality of PET images in both phantom and clinical studies. We hope that this study will contribute to the growing body of knowledge on anatomically guided PET reconstruction and its potential clinical application in brain PET/MR examinations.

## 2 Methods

### 2.1 Algorithm Implementation

We implemented parallel level sets (PLS), a penalized expectation maximization method proposed by Ehrhardt et al. on a commercial PET/MR system (uPMR790, United Imaging Healthcare Co., Shanghai, China) (Ehrhardt et al. 2016).

The penalized‐likelihood PET reconstruction problem can be written as

$$\text{argmin}\;\sum_{i}\bar{y_{i}}\left(u\right)-y_{i}\log\bar{y_{i}}\left(u\right)+\mathrm{\beta P}\left(u|v\right)$$

where $y_{i}$ are measured coincidences for line of response i, u is the PET image. The penalization term is written as

$$P\left(u|v\right)=\int_{\Omega }\left(\alpha^{2}+{\left|\nabla u\left(x\right)\right|}^{2}-{\left\langle \nabla u\left(x\right),\xi \left(x\right)\right\rangle }^{2}\right)$$

Where v is the MR prior image, α is a smoothing parameter and $\xi \left(x\right)=\nabla v\left(x\right)/{\left({\left|\nabla v\left(x\right)\right|}^{2}+\eta^{2}\right)}^{1/2}$ is the normalized MR image with edge threshold parameter η. Both α and η were optimized to fixed values. The prior term in the equation leverages the structural similarity between MR and PET, reducing the regularization term at edges shared by the two modalities. The MR prior image used in this study was a T1‐weighted 3D gradient‐echo series. The penalization strength β, which can be adjusted by the user to controls the smoothness of the image.

### 2.2 Phantom Study

**FIGURE 1:**
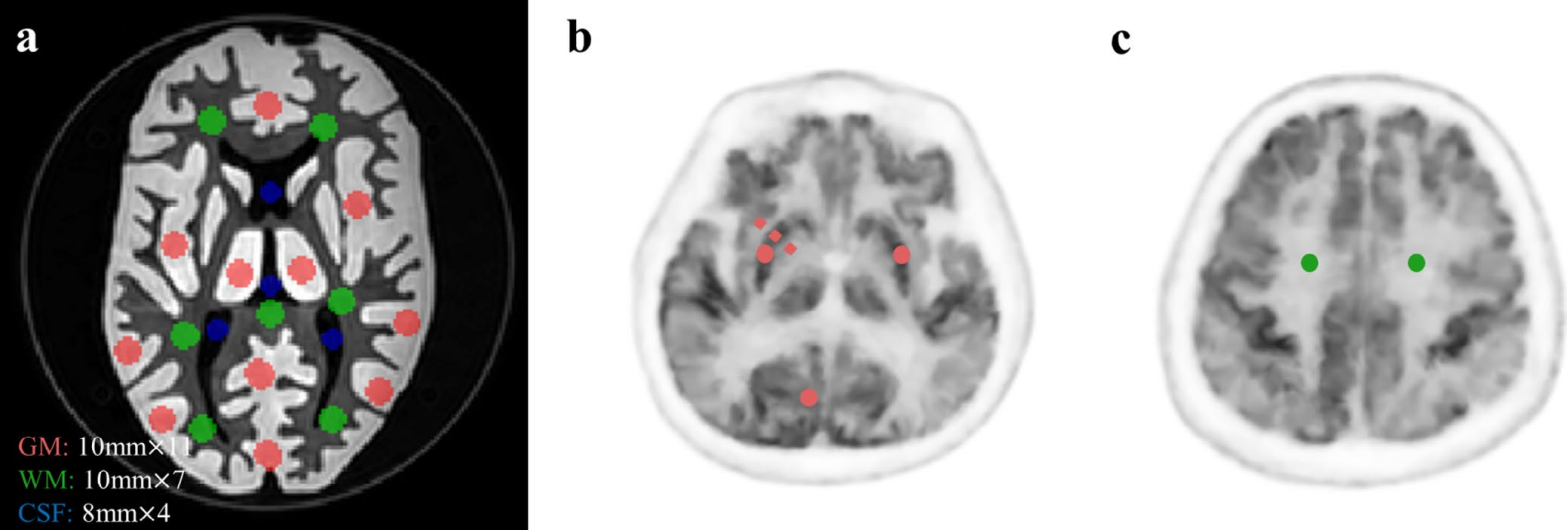
ROI placement in phantom and clinical studies for brain tissue analysis. The image depicts the locations of the ROIs used for quantitative analysis in the phantom study (a) and clinical study (b, c). The green solid circles represent the ROIs placed in the brain's white matter, while the red solid circles represent the ROIs placed in the brain's gray matter. The blue solid circles represent the ROIs placed in the cerebrospinal fluid. ROIs, regions of interests.

A Hoffman phantom was used to evaluate the image quality and quantitation accuracy of different doses at different numbers of iterations and penalization factors. The Hoffman phantom was filled with 2.26 mCi ^18^F‐FDG and scanned for 10 min. The listmode data was subsequently randomly cut to 50%, 25%, and 12.5% of the total data to represent count statistics of lower doses. PET images were reconstructed using two methods: the standard Ordered Subsets Expectation Maximization (OSEM) algorithm, which employed a Gaussian filter with a full width at half maximum (FWHM) of 3 mm, and our proposed MR‐ guided PET reconstruction method (MRg) with a penalization factors of *β* = 2, 1, 0.5. The common reconstruction parameters for both methods were as follows: 20 subsets, 8 iterations (results of each iterations were saved separately), voxel size of 1.2 × 1.2 × 1.2 mm^3^. Both reconstructions utilized time‐of‐flight (TOF), point spread function (PSF), and all necessary corrections. To evaluate the images on a quantitative basis, regions of interest (ROIs) of the gray matter (GM), white matter (WM) and cerebrospinal fluid (CSF) were drawn on a central slice of the phantom as described by Leemans et al. shown in Figure 1a (Leemans et al. 2015).

The contrast recovery coefficient (CRC) was calculated as:

$$\mathrm{CRC}=\frac{\frac{\mathrm{GM}}{\mathrm{WM}}-1}{4-1}$$

And relative noise was assessed through the coefficient of variance (COV) of the WM, calculated as:

$$\mathrm{COV}=\frac{{\mathrm{WM}}_{\mathrm{SD}}}{{\mathrm{WM}}_{\text{mean}}}$$

GM‐WM contrast was calculated as:

$$\mathrm{GM}-\mathrm{WM}\;\text{contrast}=\frac{{\mathrm{GM}}_{\text{mean}}}{{\mathrm{WM}}_{\text{mean}}}$$

GM‐CSF contrast was calculated as:

$$\mathrm{GM}-\mathrm{CSF}\;\text{contrast}=\frac{{\mathrm{GM}}_{\text{mean}}-{\mathrm{CSF}}_{\text{mean}}}{{\mathrm{GM}}_{\text{mean}}}$$

### 2.3 Clinical Study

#### 2.3.1 Clinical Information

All procedures performed in studies were in accordance with the Declaration of Helsinki. Thirty patients (age: 56.3 ± 17.5, female/male: 14/16) without any known brain tumor, two patients with diffuse large B‐cell lymphoma (DLBCL) (age: 67.5 ± 6.5, female/male: 1/1), 18 patients with Parkinson's disease (PD) (age: 63.9 ± 9.5, female/male: 10/8) and 1 patient with cavernous angioma (age: 45, male) were retrospectively enrolled in this study. These patients were referred to Shanghai Renji Hospital from June 2021 to November 2023. Clinical trial numbers are not applicable. The clinical information is summarized in Table 1.

**TABLE 1:** Clinical information.

Parameter	Patients without brain lesions	Patients with DLBCL	Patients with PD	Patients with cavernous angioma
Number of patients	30	2	18	1
Gender (female/male)	14/16	1/1	10/8	0/1
Age (years)	56.3 ± 17.5	67.5 ± 6.5	63.9 ± 9.5	45.0
Weight (kg)	68.0 ± 13.8	60.0 ± 0	58.9 ± 9.2	88.0
Height (m)	1.68 ± 0.06	1.60 ± 0.03	1.65 ± 0.08	1.80
Dose (mCi)	5.97 ± 1.15	4.95 ± 0.07	5.64 ± 0.76	9.35
Post‐injection (min)	44.6 ± 19.8	36.1 ± 3.2	39.1 ± 13.7	48.7
Scan duration (min)	20	40	40	6

Abbreviations: DLBCL, diffuse large B cell lymphoma; PD, Parkinson's disease.

#### 2.3.2 Image Acquisition

Before the ^18^F‐FDG injection, all patients had fasted for at least 6 h with the blood glucose level less than 10.0 mmol/L. PET/MR images were acquired 41.8 ± 16.3 min after the ^18^F‐FDG injection using an integrated PET/MR scanner (uPMR790, United Imaging Healthcare Co., Shanghai, China) from Renji Hospital and the mean ^18^F‐FDG injection dose was 5.89 ± 1.12 mCi. Reconstruction parameters were: 20 subsets, 6 iteration, 1.2 × 1.2 × 1.2 mm^3^ voxel size, with TOF, PSF and all corrections. The OSEM reconstructed images were post filtered by a Gaussian filter with FWHM of 3 mm, and the proposed MRg with a penalization factor of *β* = 2. As for the anatomical prior image, T1—weighted gradient echo (GRE) MR sequence was acquired with the following parameters: repetition time (TR) of 7.19 ms, echo time (TE) of 3.0 ms, inversion time (TI) of 750 ms, a flip angle of 10°, and an in—plane resolution of 512 × 460.

#### 2.3.3 Image Quality Assessment for Patients Without Brain Lesions

Thirty patients without brain lesions were included for image quality assessment ensure comparable noise level, PET data were cut to 20 min, which is a typical PET/MR scanning scenario in our hospital. Regions of interests (ROIs) were placed on the gray matter and white matter as shown in Figure 1b,c. For images using OSEM and the proposed method, several quantitative parameters were calculated to evaluate the image contrast and the image noise level including contrast‐to‐noise ratio CNR $=\frac{{\mathrm{GM}}_{\text{mean}}-{\mathrm{WM}}_{\text{mean}}}{{\mathrm{WM}}_{\mathrm{SD}}}$ and $\mathrm{COV}=\frac{{\mathrm{WM}}_{\mathrm{SD}}}{{\mathrm{WM}}_{\text{mean}}}$. Edge sharpness index was computed as the maximum slope of count profiles, which were obtained perpendicularly to the WM‐GM interfaces of the striata and occiput (dashed line in Figure 1b). The sharpness index values and are expressed in percentage of the maximal count value of the line (Salvadori et al. 2019).

For visual analysis, two experienced nuclear medicine physicians evaluated the OSEM images and the MRg images using a 5‐point Likert Scale in three dimensions: contrast, noise level, and edge sharpness, with one representing the lowest quality and five representing the highest quality. The clinical information and the reconstruction algorithm were concealed from the physicians during the evaluation process.

#### 2.3.4 Image Quality Assessment for Patients With Neurological Diseases

To test the feasibility of the MRg technology in different types of neurological diseases, two patients with DLBCL (brain tumors with high FDG uptake), 18 patients with PD (increased uptake of FDG in the bilateral putamen) and one patient with cavernous angioma (decreased uptake of FDG in the tumor) were included in this study. The data was reconstructed with OSEM and MRg algorithm, respectively, using full data to achieve best image quality.

For patients with DLCBL, the ROIs of brain lesions and background (bilateral centrum semiovale) were manually drawn by two experienced nuclear medicine physicians. The signal‐to‐background ratios ($\mathrm{SBR}=\frac{{\mathrm{SUV}}_{\max}\left(\text{lesion}\right)}{{\mathrm{SUV}}_{\text{mean}}\left(\text{background}\right)}$), signal‐to‐noise ratios ($\mathrm{SNR}=\frac{{\mathrm{SUV}}_{\max}\left(\text{lesion}\right)}{{\mathrm{SUV}}_{\mathrm{SD}}\left(\text{background}\right)}$), contrast‐to‐background ratios ($\mathrm{CBR}=\frac{{\mathrm{SUV}}_{\text{mean}}\left(\text{lesion}\right)-{\mathrm{SUV}}_{\text{mean}}\left(\text{background}\right)}{{\mathrm{SUV}}_{\text{mean}}\left(\text{background}\right)}$) and contrast‐to‐noise ratios ($\mathrm{CNR}=\frac{{\mathrm{SUV}}_{\text{mean}}\left(\text{lesion}\right)-{\mathrm{SUV}}_{\text{mean}}\left(\text{background}\right)}{{\mathrm{SUV}}_{\mathrm{SD}}\left(\text{background}\right)}$) were evaluated for each brain lesions.

For patients with PD, FreeSurfer software (https://surfer.nmr.mgh.harvard.edu/) was utilized to segment the bilateral putamen, the bilateral cerebellum cortex and the bilateral cerebellum white matter. This segmentation was performed based on the 3D T1‐weighted images using the freesurfer subcortical aseg atlas. The four brain regions of the bilateral cerebellum cortex and the bilateral cerebellum white matter were merged into a single cerebellum region using the DPABI software (Data Processing & Analysis for Brain Imaging, https://rfmri.org/DPABI) (Yan et al. 2016). The OSEM and the MRg PET images were co‐registered to the corresponding 3D T1‐weighted images using the Statistical Parametric Mapping software (SPM12, https://www.fil.ion.ucl.ac.uk/spm/software/spm12) and the standardized uptake value ratios (SUVRs) of the bilateral putamen were calculated based on the normalization of the individual SUVs of bilateral putamen to the SUV of the cerebellum. In addition, the contrast‐to‐noise ratios ($\mathrm{CNR}=\frac{{\text{SUVR}}_{\text{mean}}\left(\text{putamen}\right)-{\text{SUVR}}_{\text{mean}}\left(\text{centrum semiovale}\right)}{{\text{SUVR}}_{\mathrm{SD}}\left(\text{centrum semiovale}\right)}$) of the bilateral putamen were calculated.

#### 2.3.5 Statistical Analysis

All statistical analyses were performed using MATLAB R2019a (MathWorks, Natick, Massachusetts). Interobserver agreement was calculated using the intraclass correlation coefficient (ICC), with ICC > 0.75 indicating good agreement. The quantitative and qualitative parameters for images using OSEM and MRg were compared using the Wilcoxon signed‐rank test. *p* value less than 0.05 was considered significant.

## 3 Results

### 3.1 Phantom Study

**FIGURE 2:**
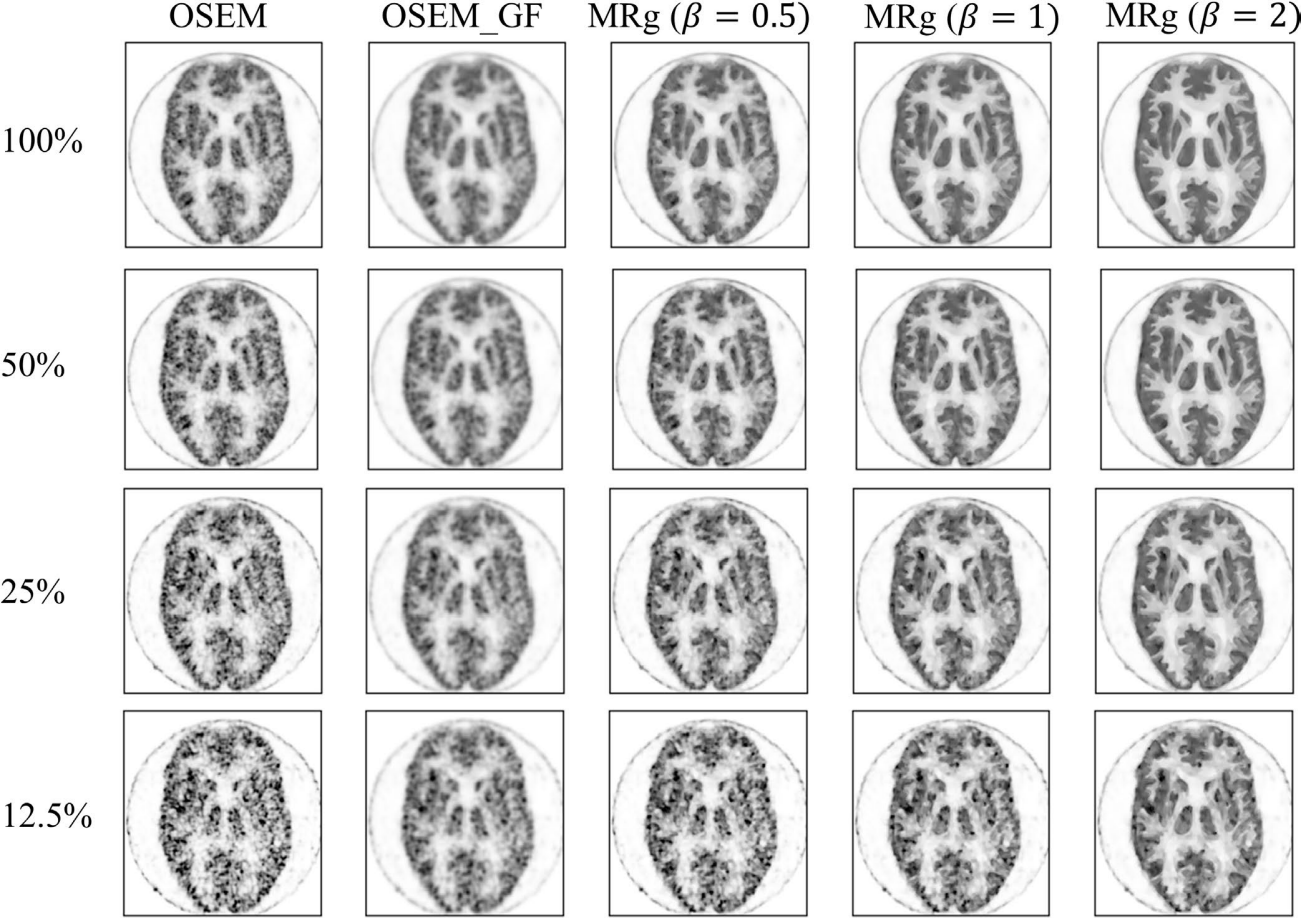
Comparison of PET image quality in hoffman phantom with different doses, reconstruction algorithm, filtering, and penalization factors (β). Each row represents images reconstructed under different doses, and each column represents different types of reconstruction techniques. Visually, it is evident that MRg (β = 2) significantly improves the signal‐to‐noise ratio and tissue contrast of the images compared to OSEM, OSEM_GF, MRg (β = 0.5), and MRg (β = 1). This improvement is most pronounced when the injection dose is only 12.5% of the full dose. The β refers to the regularization parameter or shrinkage parameter, which controls the amount of regularization applied to the model. OSEM ordered subsets expectation maximization. GF, Gaussian filtering; MRg, MR‐guided PET reconstruction.

Figure 2 shows sample slices of the reconstructed Hoffman phantom images. For OSEM reconstruction, image quality degraded rapidly with doses less than 50%. While Gaussian smoothing reduced noise levels, it resulted in a loss of contrast between different tissue types and a blurred image. In contrast, MRg images not only preserved edges but also effectively reduced noise. Additionally, for low‐dose data, larger penalization factors were required to optimize image quality.

**FIGURE 3:**
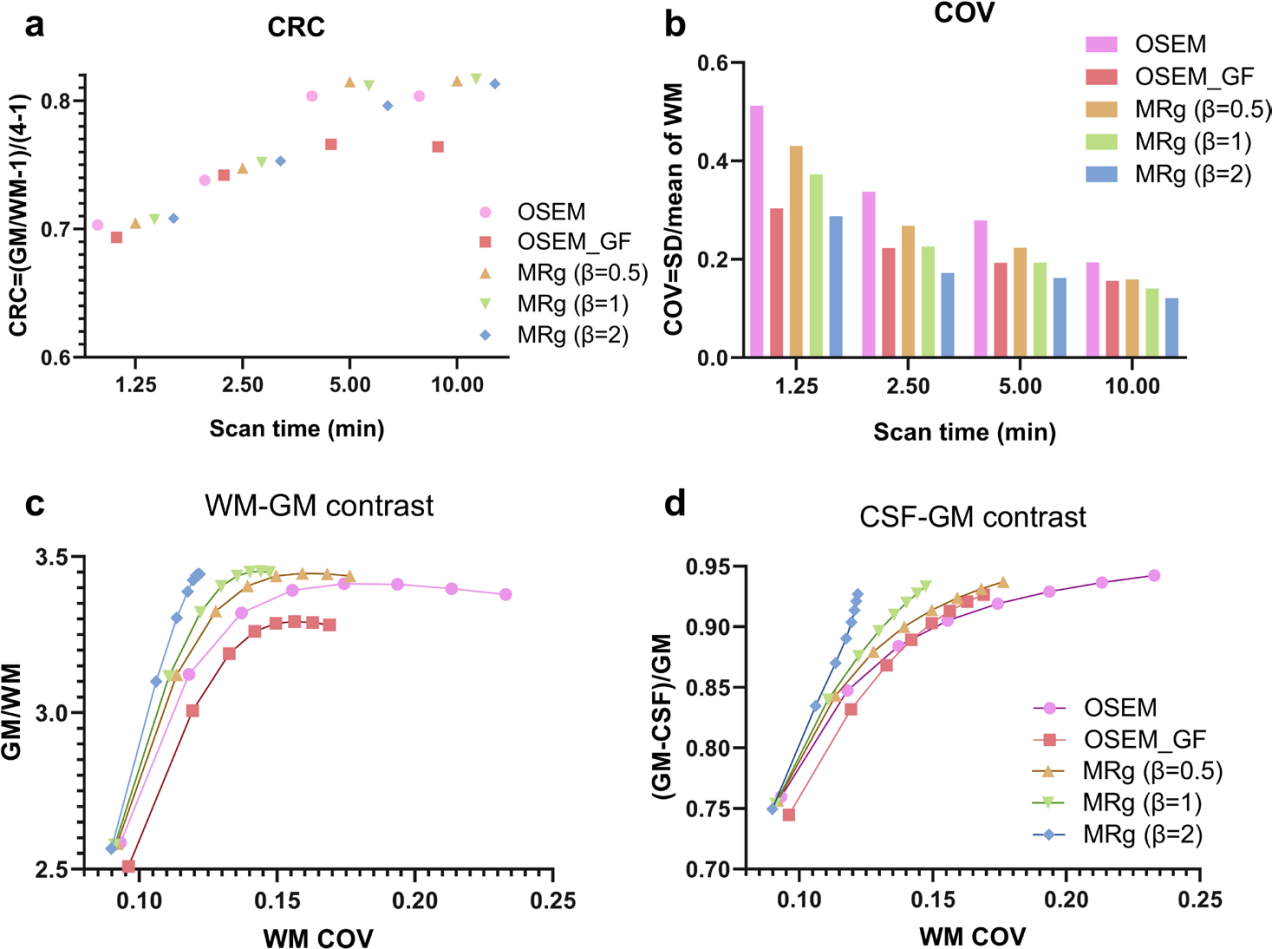
Quantitative results of the phantom study. (a–d) Demonstrate the comparison of various image evaluation parameters (CRC, COV, GM to WM contrast, and the GM to CSF contrast) among five different reconstruction algorithms. MRg (β = 2) significantly improves all these parameters compared to OSEM. COV, coefficient of variance; CRC, contrast recovery coefficient; CSF, cerebrospinal fluid; GF, Gaussian filtering; GM, gray matter; MRg, MR‐guided PET reconstruction; OSEM ordered subsets expectation maximization; SD, standard deviation; WM, white matter.

The quantitative results are shown in Figure 3. For OSEM and MRg reconstruction with 6 iterations, Figure 3a showed that MRg resulted in comparable or higher CRC compared to OSEM without Gaussian filtering (GF). Meanwhile, GF led to a decreased image contrast. For noise level evaluated by COV, MRg images presented lower COV compared to OSEM without GF, and the difference was more pronounced for images with lower doses, as shown in Figure 3b. GF in OSEM could reduce noise, but it came at the cost of losing CRC. Figure 3c and Figure 3d demonstrated that the GM to WM contrast and the GM to CSF contrast improved with the increasing iteration number. MRg of all β resulted in higher contrast in the same noise level or similar contrast with less noise compared to OSEM with or without GF.

### 3.2 Clinical Study

#### 3.2.1 Image Quality Assessment for Patients Without Brain Lesions

**FIGURE 4:**
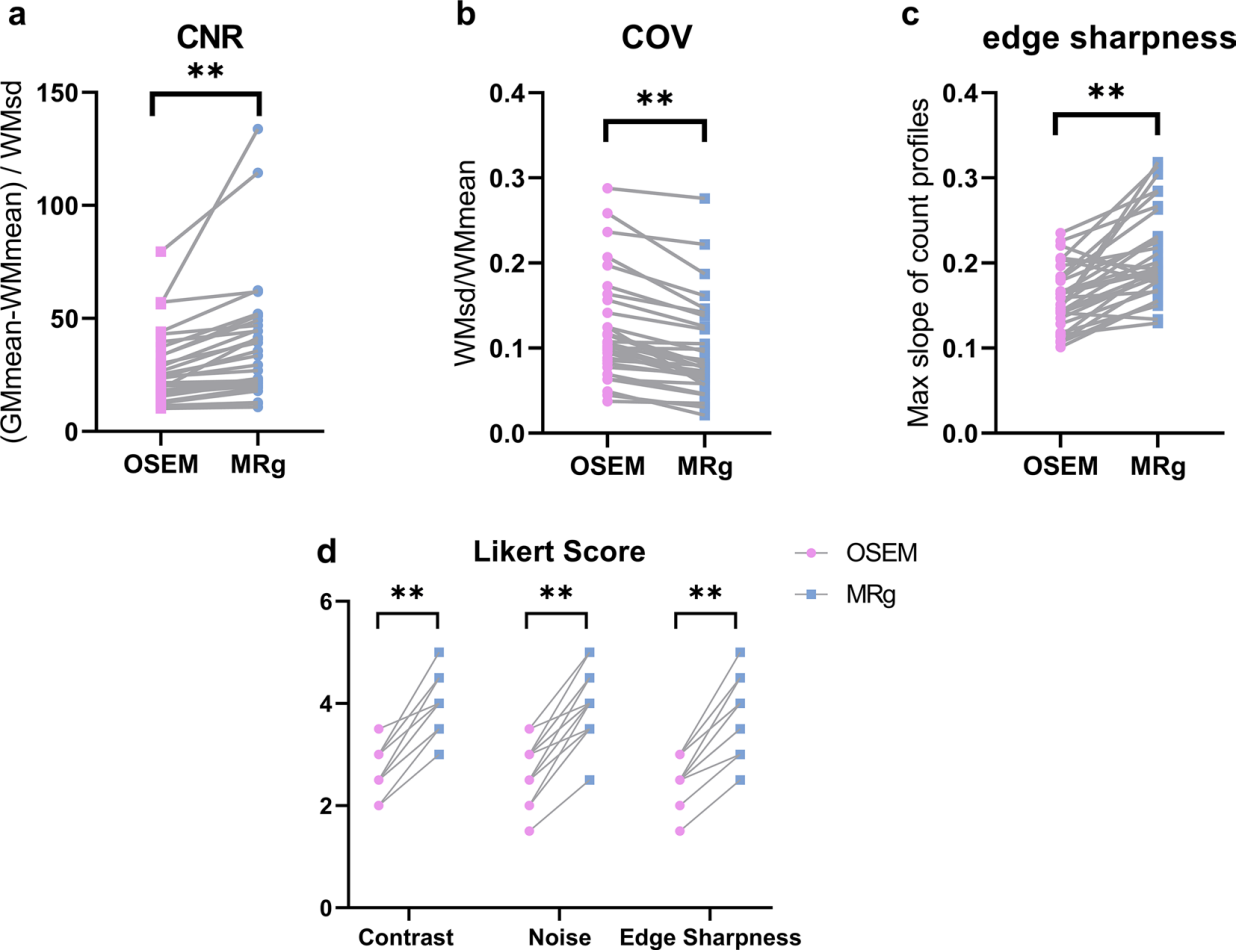
Quantitative and qualitative clinical study results for patients without brain lesions. (a–c) Demonstrates the differences in CNR, COV, and edge sharpness between the OSEM and MRg algorithms. MRg achieved better scores compared to OSEM(**p < 0.001). (d) Shows the differences in visual scores for contrast, noise level, and edge sharpness between the OSEM and MRg algorithms (**p < 0.001). CNR, contrast to noise ratio; COV, coefficient of variance; MRg, MR‐guided PET reconstruction; OSEM, ordered subsets expectation maximization.

The quantitative results are presented in Figure 4a–c, which show that MRg had higher CNR and edge sharpness and lower background noise compared to OSEM. By introducing the MR prior, CNR was enhanced by 35.6% ± 29.5%, edge sharpness was enhanced by 33.8% ± 30.8%, and COV was reduced by 21.8% ± 13.7%. These differences were statistically significant, with a *p* value of less than 0.001.

For visual analysis, the values of showed good inter‐observer agreement for all parameters (ICC > 0.75). The qualitative scores of the two physicians were averaged for comparison; the results are shown in Figure 4d. The image quality scores of the MRg images were significantly improved compared to the OSEM images (*p* < 0.001) (Table 2).

**TABLE 2:** Qualitative results of the clinical study for patients without brain lesions.

Parameter	OSEM	MRg	*p*
Contrast	2.65 ± 0.33	4.15 ± 0.51	*p* < 0.001
Noise	2.70 ± 0.45	4.10 ± 0.55	*p* < 0.001
Edge sharpness	2.65 ± 0.35	3.93 ± 0.57	*p* < 0.001

Abbreviations: MRg, MR‐guided PET reconstruction; OSEM, ordered subsets expectation maximization.

#### 3.2.2 Image Quality Assessment for Patients With Neurological Diseases

**FIGURE 5:**
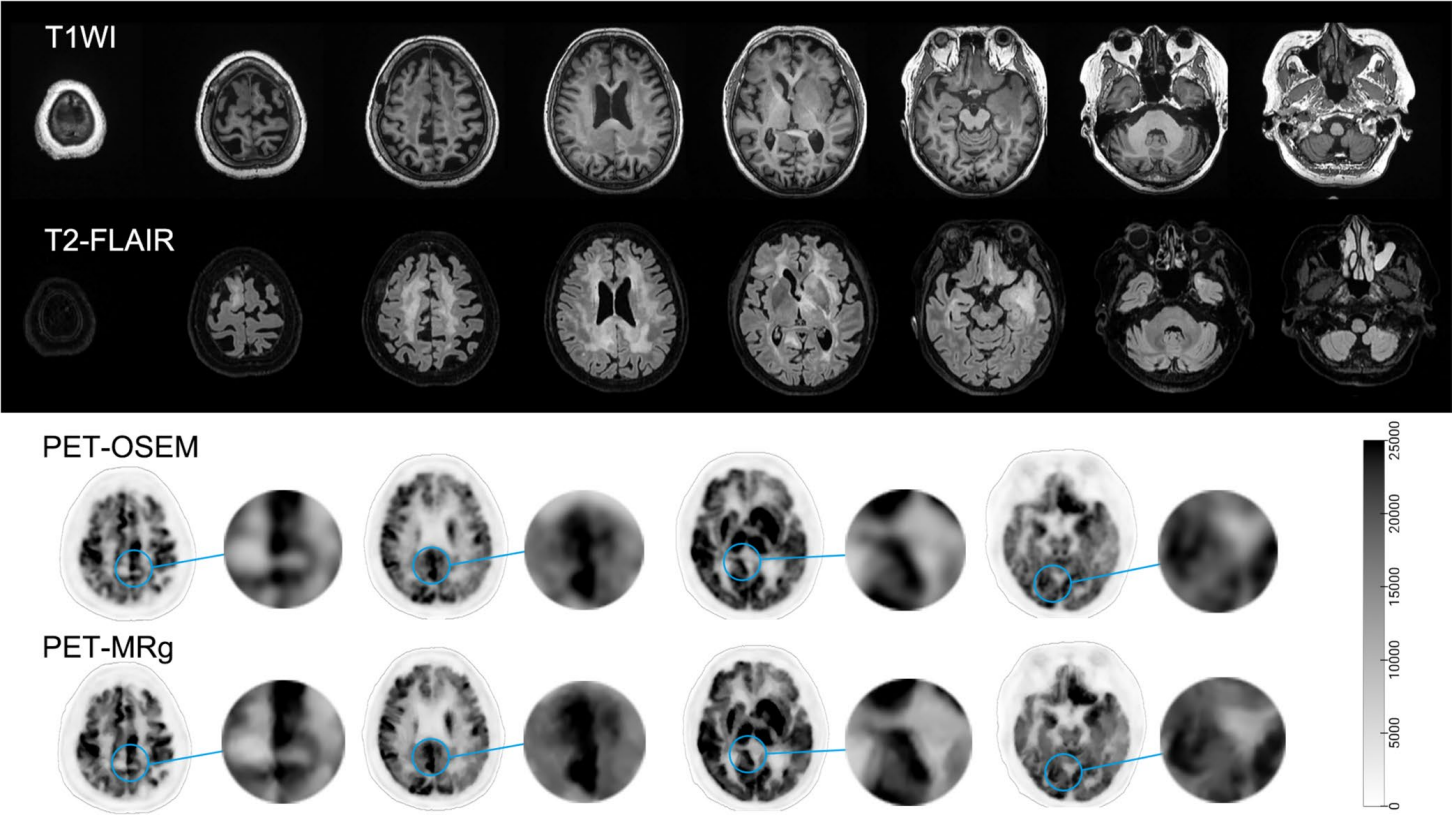
MRg and OSEM PET in a 74‐year‐old female with DLBCL. The MR T1WI (first row) and T2‐FLAIR (second row) images reveal multiple abnormal signal patches in the bilateral cerebral white matter and cortex, predominantly in the left inferior frontal gyrus and left basal ganglia. Visual analysis indicates that the PET‐MRg images have a superior signal‐to‐noise ratio and clearer tissue boundaries compared to the PET‐OSEM images. DLBCL, diffuse large B‐cell lymphoma; FDG, fluorodeoxyglucose; FLAIR, fluid attenuated inversion recovery; MR, magnetic resonance; MRg, magnetic resonance guided PET reconstruction; OSEM, ordered subset expectation maximization; PET, positron emission tomography; T1WI, T1 weighted image.

**FIGURE 6:**
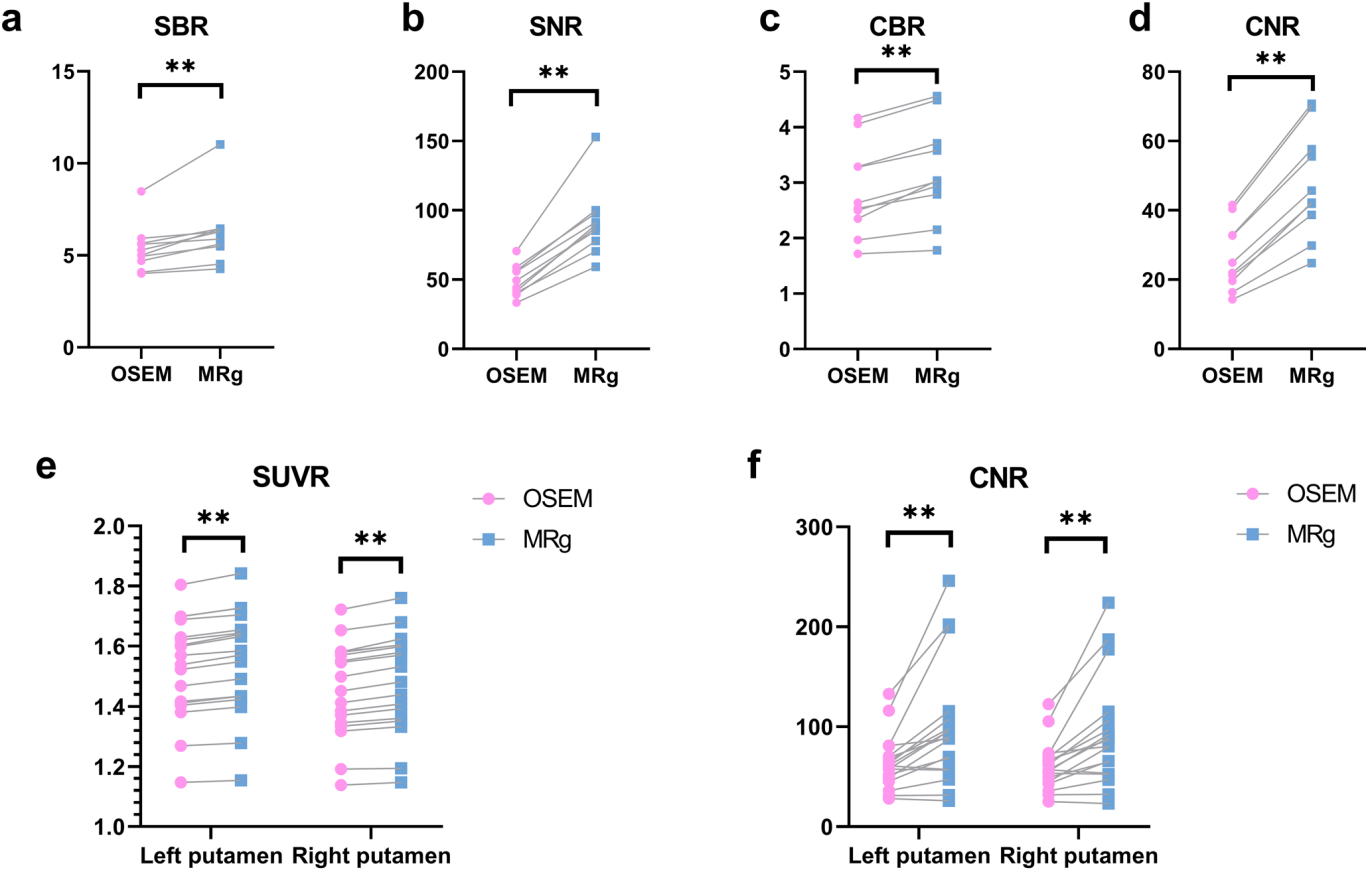
Quantitative results of the clinical study for patients with DLBCL and PD. (a–d) Demonstrate the differences of SBR, SNR, CBR, and CNR of lesions between the OSEM and MRg algorithms in patients with DLBCL (**p < 0.001). (e, f) Show the differences of SUVR and CNR of bilateral putamen between the OSEM and MRg algorithms in patients with PD (**p < 0.001). CBR, contrast‐to‐background ratio; CNR, contrast‐to‐noise ratio; DLBCL, diffuse Large B‐cell lymphoma; MRg, magnetic resonance‐guided PET reconstruction; OSEM, ordered subsets expectation maximization; PD, Parkinson's disease; SBR, signal‐to‐background ratio; SNR, signal‐to‐noise ratio; SUVR, standardized uptake value ratio.

In the case of the two patients diagnosed with DLBCL, a total of 10 lesions were detected by experienced nuclear medicine physicians. Figure 5 illustrates the OSEM and the MRg PET images of a 74‐year‐old patient with DLBCL. Visual analysis suggests that the MRg images have better SNR and clearer tissue boundaries than the OSEM images. Remarkably, all four quantitative metrics of the lesions exhibited significant improvements when using the MRg algorithm (SBR enhancement of 15.0% ± 8.6%, SNR enhancement of 86.0% ± 18.7%, CBR enhancement of 13.0% ± 6.6% and CNR enhancement of 81.0% ± 13.0%). The results are shown in Figure 6a–d.

**FIGURE 7:**
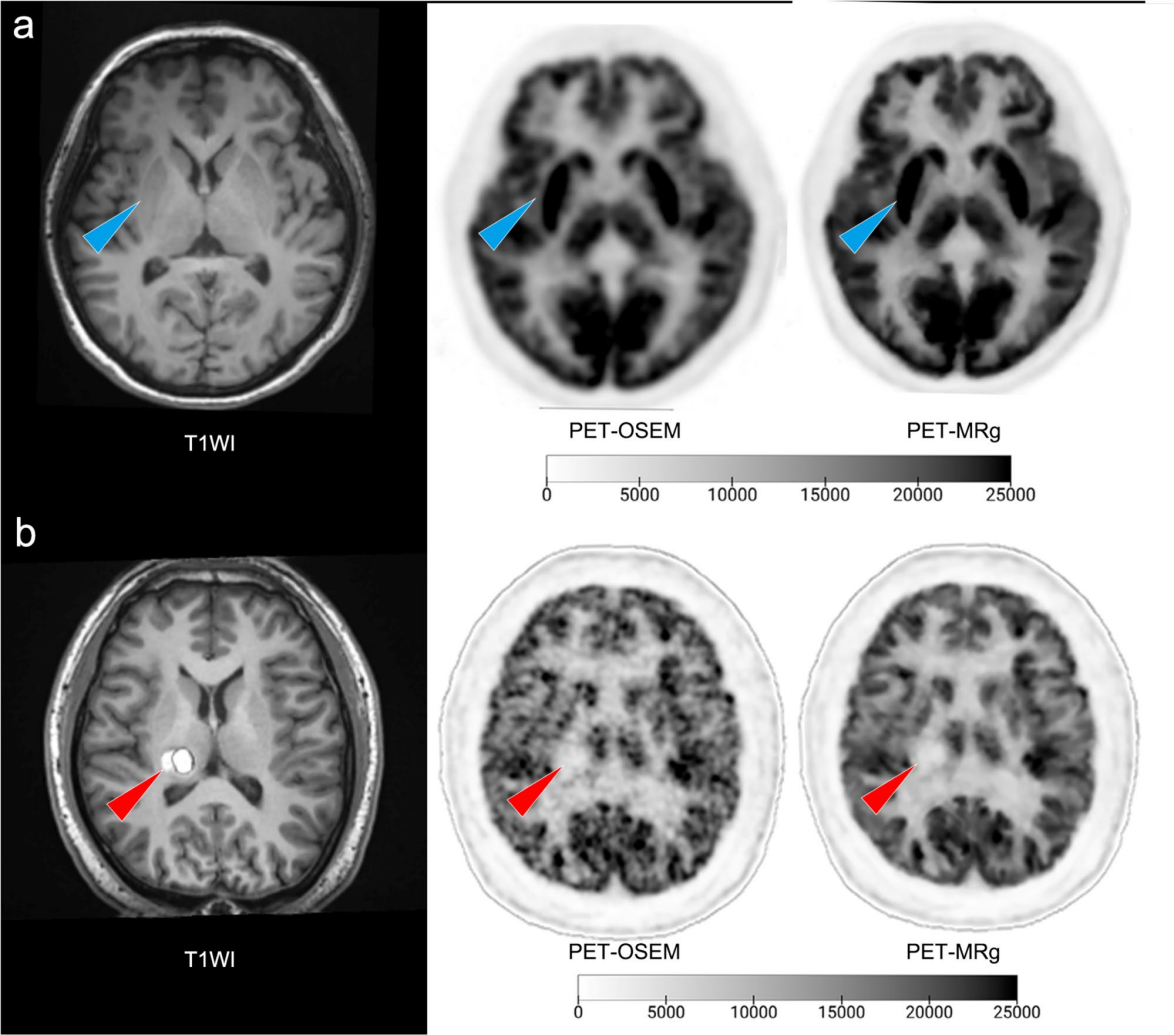
Example images of a patient with PD and a patient with cavernous angioma. (a) Shows the MR T1WI, PET‐OSEM, and PET‐MRg images of a 77‐year‐old male patient with Parkinson's disease visual analysis indicates that the PET‐MRg image is clearer than the PET‐OSEM images, with more uniform FDG metabolism and better‐defined boundaries in the putamina (blue arrow). (b) Depicts the MR T1WI, PET‐OSEM, and PET‐MRg images of a 45‐year‐old male patient with cavernous angioma. The PET‐MRg image depicts reduced metabolism in the lesion (red arrow) compared to normal brain white matter with a more precise identification of the lesion boundaries compared to PET‐OSEM. FDG, fluorodeoxyglucose; MR, magnetic resonance; MRg, magnetic resonance‐guided PET reconstruction; OSEM, ordered subset expectation maximization; PD, Parkinson's disease; PET, positron emission tomography; T1WI, T1 weighted image.

For patients diagnosed with PD, the T1WI MR image, OSEM, and MRg PET images of a representative patient are illustrated in Figure 7a. The MRg image exhibits greater clarity than the PET‐OSEM images, with more uniform FDG accumulation and better‐defined boundaries in the putamina. All quantitative metrics of the bilateral putamen significantly improved using the MRg algorithm (*p* < 0.001). MRg increased the SUVR of the left putamen by 1.48% ± 0.47% and the right putamen by 1.59% ± 0.56%, and the CNR of the left putamen by 46.4% ± 40.1% and the right putamen by 46.6% ± 40.1%. The results are shown in Figure 6e,f.

For the patient diagnosed with cavernous angioma, the T1WI MR image, OSEM, and MRg PET images are shown in Figure 7b. In comparison to OSEM, MRg exhibits superior SNR and enhanced contrast between WM and GM in the image. Interestingly, although the tumor exhibited high signal intensity in the T1WI image, the application of MRg reconstruction avoided potential misinterpretation of a hypermetabolic tumor. It offered a more clearly defined tumor margin and a reduced level of image noise, thereby enhancing the diagnostic capabilities of PET‐MRg over conventional OSEM.

## 4 Discussion

In this work, we implemented an MR‐guided PET reconstruction algorithm into an integrated PET/MR system and evaluated its performance with phantom and clinical experiments. We observed that the use of anatomical information from MR results in substantial improvement of PET image quality both visually and quantitatively.

The novelty of this study lies in applying the MRg technique to the clinical setting of an integrated PET/MR system. Although various MRg methods have been proposed in the literature, there has been limited clinical experience with this approach (Schramm et al. 2021). Among the various MRg reconstruction methodologies published, we chose PLS due to its superior performance in terms of bias and quantification accuracy (Schramm et al. 2018). It also has better robustness when dealing with boundary discrepancies between MR and PET images. As Ehrhardt et al. reported, prior information does not distort the unique features of PET images, nor does it introduce artifacts related to MR‐specific features (Ehrhardt et al. 2016). In this study, the case of the patient with cavernous angioma confirmed that this algorithm avoids generating an artificially hypermetabolic artifact on PET images, even when the lesion is clearly visible on the MR T1WI image.

The Hoffman phantom, which is designed to mimic the brain's FDG uptake, served as a reference point for our determination of the reconstruction parameters. In the phantom study, MRg reconstruction yielded CRC comparable to OSEM, better GM to WM contrast and GM to CSF contrast, and significantly lower COV than OSEM, especially in lower doses. Notably, the choice of penalization factor substantially impacts the outcomes and should be selected carefully depending on acquisition settings and the radiologists' preference. For instance, as shown in Figure 3a,b, 12.5% dose images require β=2 to attain CRC as high as OSEM and COV comparable to Gaussian‐filtered OSEM, whereas for 50% dose images, β=1 were good enough. Our non‐tumor patient data had comparable counting statistics as the full‐dose phantom data, and therefore we were convinced that β=1~2 was suitable for our clinical data. Considering that *β* = 2 demonstrated a more pronounced effect, and that *β* = 2 proved to be more robust when handling lower‐dose situations, we chose β=2 in our clinical studies to explore the benefits of MRg.

In the image quality assessment studies, MRg images were rated as more visually appealing and quantitatively demonstrated higher contrast, lower noise level, and higher image sharpness. For patients with brain tumors, high‐quality FDG‐PET images are crucial because they provide prognostic information to differentiate benign and malignant tumors and guide biopsies and surgical procedures (Binneboese et al. 2021; Pietrzak et al. 2021; Colavolpe et al. 2012; Verger and Langen 2017). FDG‐PET has been extensively and profoundly researched in the field of DLBCL. DLBCL typically exhibits a very pronounced accumulation of the FDG radiotracer on PET imaging. Following effective treatment, there is a marked decrease in the FDG uptake in DLBCL tissues. Therefore, the image quality of FDG‐PET images is crucial for diagnosis and prognostic assessment for DLBCL diseases (Ferdová et al. 2017). In our study, we found that several small lesions that resembled image noise in OSEM were likely to be overlooked. In the MRg image, they have a more precise boundary and become more distinct from noise. The lesions' signal/contrast‐to‐background/noise ratios were significantly elevated in the MRg images, making the small lesions more visible to physicians. This advancement provides strong support for selecting biopsy regions of brain lesions and ensuring precise diagnostic outcomes for patients. Biopsies were conducted on the two patients under the guidance of FDG‐PET imaging, and subsequent histopathological examination confirmed the diagnosis of DLBCL in both cases. This may indicate that MRg can potentially improve the detection of small lesions in brain tumor patients or at least enhance the confidence of physicians in diagnosing.

FDG PET imaging is an essential diagnostic tool for neurological disorders such as Parkinson's disease. Early detection of metabolic changes is crucial for PD diagnosis, as the abnormal pattern and extent of FDG uptake in specific brain regions (e.g., putamen) may indicate the rate of motor decline, often present before clinical symptoms emerge (Matthews et al. 2018). Not only is FDG PET a prognostic indicator, but it also aids in distinguishing Parkinson's disease from other Parkinsonian syndromes and helps classify Parkinson's disease subtypes, which may have varying pathologies and treatment responses (Meyer et al. 2017; Matthews et al. 2018; Walker et al. 2018; Booth et al. 2022). It is also valuable for monitoring disease progression and evaluating the effectiveness of therapeutic interventions (Matthews et al. 2018). In China, the utilization of CFT‐PET imaging is not as prevalent as that of FDG PET, thereby highlighting the heightened significance of obtaining high‐quality FDG PET images for the diagnosis and treatment evaluation of Parkinson's disease. Therefore, achieving clear FDG PET images is vital for accurate neurological assessments. For nuclear medicine physicians, discerning FDG PET images indicative of classic Parkinson's disease or those of a healthy individual is generally a straightforward task. However, in scenarios where the FDG PET images of PD patients are ambiguous, lying at the boundary between typical and normal patterns, the presence of a clear and detailed FDG PET image can substantially boost the physician's confidence and accuracy in making a diagnosis. In this study, we observed that by performing MRg reconstruction, the SUVR and CNR for both left and right putamen increased and the shape of putamen became clearer compared to routine OSEM. This suggests that this advanced reconstruction method may be beneficial for the diagnosis and prognosis of PD. This research has several limitations. First, clinical scenarios of neurological disorders were limited, and the sample size was relatively small, with only 2 patients with DLBCL, 18 patients with PD, and one patient with cavernous angioma. Different pathologies may affect the image pattern and quality and potentially influence the behavior of the MRg algorithm. Although *β* = 2 may offer a baseline effect of the MRg algorithm, the optimal value of the penalization factor remains to be determined carefully for special cases. Expanding the range of clinical scenarios and increasing the patient sample size in future studies is essential to validate the stability of the proposed method. Second, although we demonstrated the enhancements in PET image quality and the conspicuity of tumors as well as bilateral putamen, there is a lack of direct evidence to show the impact of the MRg algorithm on clinical diagnosis. Further studies are needed to evaluate the algorithm's performance in clinical diagnostic settings. Thirdly, as the MRg algorithm is applicable to various PET radiotracers, parameter fine‐tuning for each specific tracer according to clinical scenario is necessary to achieve optimal results. Future research is needed to explore the performance of the MRg algorithm with different PET tracers.

## 5 Conclusion

In this work, we observed that the MR‐guided PET reconstruction technique can effectively enhance the quality of brain PET images. The improvement in image quality is crucial for early diagnosis of diseases, accurate assessment of treatment effectiveness, and reduction of misdiagnosis rates. With ongoing advancements in PET image reconstruction technology, we anticipate obtaining more precise and reliable PET images in the future, thereby further improving the quality of medical services and patient outcomes.

## Author Contributions

Hongda Shao and Gan Huang contributed equally as first authors. Hongda Shao and Gan Huang were primarily responsible for the acquisition, analysis, and interpretation of data. Yue Wang, Yan Zhang, Qiaoyi Xue, and Jiaxin Hao participated in the data collection and analysis. All authors were involved in drafting the article or revising it critically for important intellectual content. Jianjun Liu, as the corresponding author, was responsible for the conception and design of the study, as well as supervising the entire research process and ensuring the final approval of the version to be submitted.

## Ethics Statement

This study was performed in line with the principles of the Declaration of Helsinki. Approval was granted by the Ethics Committee of Ren Ji Hospital, Shanghai Jiao Tong University School of Medicine (2024‐11‐8/No. LY2024‐238‐B).

## Consent

Informed consent was obtained from all individual participants included in the study.

## Conflicts of Interest

Qiaoyi Xue and Jiaxin Hao are employees of the United Imaging Healthcare group.

## Data Availability

The datasets generated during and/or analyzed during the current study are available from the corresponding author on reasonable request.
